# Learning to cooperate for low-Reynolds-number swimming: a model problem for gait coordination

**DOI:** 10.1038/s41598-023-36305-y

**Published:** 2023-06-09

**Authors:** Yangzhe Liu, Zonghao Zou, On Shun Pak, Alan C. H. Tsang

**Affiliations:** 1 Department of Mechanical Engineering, The University of Hong Kong, Pokfulam Road, Hong Kong, China; 2 Sibley School of Mechanical and Aerospace Engineering, Cornell University, Ithaca, NY 14850 USA; 3 Department of Mechanical Engineering, Santa Clara University, Santa Clara, CA, 95053 USA

**Keywords:** Fluid dynamics, Applied mathematics

## Abstract

Biological microswimmers can coordinate their motions to exploit their fluid environment—and each other—to achieve global advantages in their locomotory performance. These cooperative locomotion require delicate adjustments of both individual swimming gaits and spatial arrangements of the swimmers. Here we probe the emergence of such cooperative behaviors among artificial microswimmers endowed with artificial intelligence. We present the first use of a deep reinforcement learning approach to empower the cooperative locomotion of a pair of reconfigurable microswimmers. The AI-advised cooperative policy comprises two stages: an approach stage where the swimmers get in close proximity to fully exploit hydrodynamic interactions, followed a synchronization stage where the swimmers synchronize their locomotory gaits to maximize their overall net propulsion. The synchronized motions allow the swimmer pair to move together coherently with an enhanced locomotion performance unattainable by a single swimmer alone. Our work constitutes a first step toward uncovering intriguing cooperative behaviors of smart artificial microswimmers, demonstrating the vast potential of reinforcement learning towards intelligent autonomous manipulations of multiple microswimmers for their future biomedical and environmental applications.

## Introduction

In nature, animals like fish and bird use their fluid environment—and each other—to gain advantage for their locomotion^1,2^, leading to fascinating pattern formation and adjustments of locomotory gaits observed in fish schooling and bird flocking. Such cooperative behaviors are also ubiquitous in the microscopic world, where swimming microorganisms exploit hydrodynamic interactions to enhance their locomotory performance^3,4^. Successful cooperative locomotion between microswimmers would require fine adjustments of not only their individual swimming gaits but also their spatial arrangements simultaneously. Biological microswimmers can evolve strategies to achieve such a complex coordination. For example, a pair of nearby sperm cells phase-lock and synchronize their flagellar beating patterns to swim cooperatively^5–8^. Yet, there are no evolved strategies available for cooperative locomotion of artificial microswimmers^9,10^. Moreover, strategies employed by biological microswimmers may not be directly applicable to artificial microswimmers, which have intrinsically different actuation mechanisms. Pioneering works have endowed artificial microswimmers with artificial intelligence (AI) to acquire effective locomotion strategies^11–17^. These advances prompt several general questions we set out to address here: When artificial microswimmers are equipped with adaptive decision making, what are the strategies for them to cooperate and achieve enhanced locomotion otherwise unattainable by isolated swimmers? Do these microswimmers adapt their strategy at different stages of cooperative swimming? How should the locomotory gaits of neighbouring swimmers be adjusted to exploit hydrodynamic interactions for maximizing their overall propulsion?

In this work, we present the first use of reinforcement learning (RL) to investigate cooperative locomotion of microswimmers. Recent studies have demonstrated the prowess of RL as a new approach to investigate locomotion problems in fluids. Different RL techniques have been utilized to empower simple reconfigurable microswimmers consisting of linked spheres to self-learn effective locomotory gaits based on interactions with the surrounding fluid^15,18–20^. Without any prior knowledge of locomotion at low Reynolds number (Re), these smart microswimmers are capable of evolving effective locomotory gaits to perform complex maneuvers such as targeted navigation and chemotactic responses^19,20^. Recent experimental studies have also begun to realize artificial microswimmers with control systems integrated with RL algorithms^16,21^. Other machine learning approaches have also been proposed to address locomotion problems in fish^22–27^ as well as navigation problems of self-propelled particles^11,13,14,16,17^. There are initial efforts on extending machine learning approaches to cooperative locomotion problems at high Re such as fish locomotion^23–25^. Yet, the cooperative behavior of smart artificial microswimmers remains a largely unexplored area of research.

**Figure 1:**
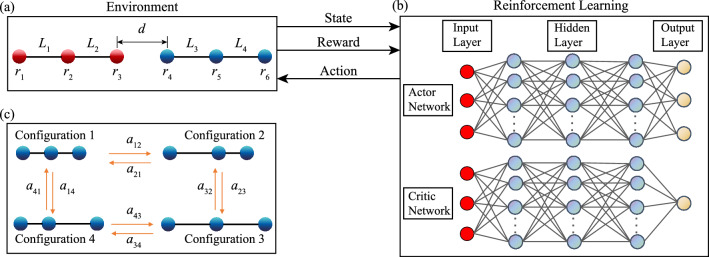
Schematics of a pair of three-sphere microswimmers with colinear arrangement and Actor-Critic neural network architecture. (**a**) Schematic of environment setup for reinforcement learning. Each swimmer consists of three rigid spheres with radius *R* and two extensible arms, and two identical swimmers are arranged colinearly. We distinguish the two swimmers by marking the spheres of the swimmer at the back as red and the spheres of the swimmer at the front as blue. The lengths of the extensible arms are denoted by $L_{i}$ (*i* = 1,2,3,4) and the positions of the spheres’ centers are denoted by ${\textbf {r}}_i$ (*i* = 1,2,...,6). The closest distance between two swimmers is denoted as *d*, which is defined as the distance between ${\textbf {r}}_3$ and ${\textbf {r}}_4$. (**b**) The deep neural network has an Actor-Critic structure, in which the Actor-network memorizes and updates the learning policy, and the Critic-network estimates a value function to evaluate the performance of the policy. (**c**) Schematic showing the transition of the swimmer’s configuration due to its actuation. The swimmer can either extend or contract one of its two links at a step and each swimmer has a total of 4 possible configurations.

Here, as a first step, we consider a simple model problem of a pair of reconfigurable microswimmers consisting of three linked spheres aligned colinearly (Fig. 1a). The locomotory gait for a single three-sphere swimmer was first studied by Najafi and Golestanian, which generate net propulsion by modulating the relative distances between the spheres^28^. The three-sphere swimmer, together with pioneering work of Purcell’s three-link swimmer^29^, represent canonical examples on how to escape the constraints of the scallop theorem and generate self-propulsion at low Re. Recent works have demonstrated how RL enables the self-learning of effective locomotory gaits of a single three-sphere swimmer^15,19,20^. Here we employ a deep neural network with an Actor-Critic structure (Fig. 1b) to investigate how RL enables two three-sphere swimmers to coordinate their motions to enhance the overall locomotory performance. The swimmer pair will learn how to exploit hydrodynamic interactions by finely adjusting their individual locomotory gaits as well as modulating relative distances between each other. We show that the swimmers approach each other initially and both swimmers eventually swim in Najafi-Golestanian’s strokes (N-G-strokes) with a constant phase mismatch between their gaits. The synchronized motions allow the swimmer pair to move together coherently with an enhanced locomotion performance unattainable by a single swimmer alone. This work constitutes a first step toward uncovering intriguing cooperative behaviors of smart artificial microswimmers.

**Figure 2:**
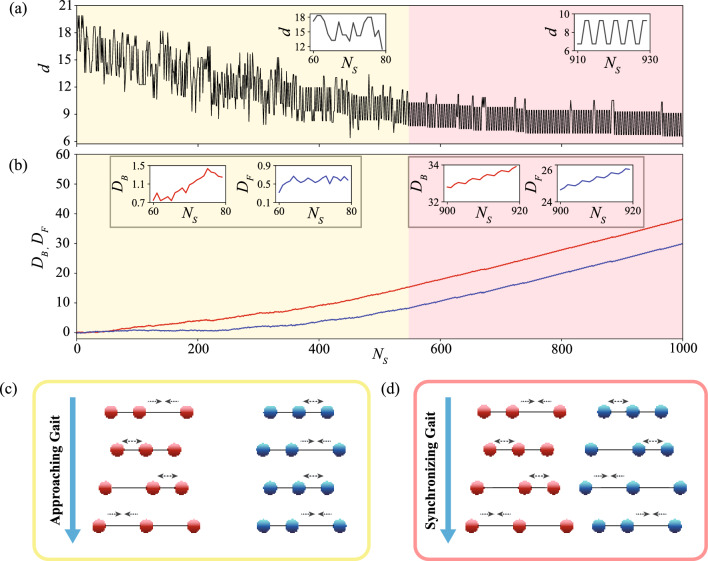
Visualization of AI-advised policy of cooperative locomotion in an isolated evaluation environment. (**a**) Change in *d* of the AI-advised policy with respect to the number of steps $N_s$. (**b**) Displacement of the swimmer at the front ($D_F$, blue line) and Displacement of the swimmer at the back ($D_B$, red line). In (**a**) and (**b**), the yellow and pink regions correspond to the approach stage and the synchronization stage, respectively. (**c**) Schematic of locomotory gaits for the approach stage, where the swimmer at the back follows N-G strokes and the swimmer at the front either generates zero net propulsion or propels with a small distance occasionally. (**d**) Schematics of locomotory gaits for the synchronization stage, where two swimmers swim cooperatively with N-G strokes at a constant phase mismatch. The swimmer at the front has a delay in 1 actuation step compared to the swimmer at the back.

## Swimmer model and deep reinforcement learning framework

### Model of a pair of reconfigurable microswimmers

We consider a pair of colinear, reconfigurable microswimmers comprising three spheres with radius *R* connected by extensible arms of negligible diameters (Fig. 1). The positions of the spheres are denoted by ${\textbf {r}}_i$ ($i= 1$ to $i= 6$) and the lengths of the arms are denoted by $L_i$ ($i= 1$ to $i= 4$). The swimmer transitions from one configuration to the other by extending or contracting one arm at a time (Fig. 1c), where we set the extended length and contracted length of the arms to be 10*R* and 6*R*, respectively. A set of effective locomotory gaits for a single three-sphere swimmer was obtained by Najafi and Golestanian^28^, which is featured by a periodic sequence of motions from configuration 1 to configuration 4 illustrated in Fig. 1c. Subsequent studies have used similar reconfigurable systems to generate net translation, rotation, and combined motion^30–37^, including a recent application of deep RL to obtain effective locomotory gaits for complex maneuvers^20^. Instead of focusing on locomotion problems of a single microswimmer as in these previous studies, here we investigate effective strategies for cooperative locomotion of a pair of reconfigurable swimmers via RL approach.

### Hydrodynamic interactions

The hydrodynamics of the reconfigurable microswimmers at low Re flow is governed by the Stokes equation ($\mu \nabla ^{2} {\textbf {u}}=\nabla p$, $\nabla \cdot {\textbf {u}}=\textbf{0}$). Here, *p*, $\mu$ and ${\textbf {u}}$ denote the pressure, dynamic viscosity, and velocity field, respectively. When the spheres are far apart from each other (i.e. in the limit of $R/L_i \ll 1$), the leading-order hydrodynamic interactions between the spheres in the fluids can be captured by the Oseen tensor^38–40^. The velocities of spheres ${\textbf {V}}_i$ and the forces acting on each sphere ${\textbf {F}}_i$ are related as

1 $${\mathbf{V}}_{i} = \sum\limits_{j = 1}^{N} {{\mathbf{H}}_{ij} } {\mathbf{F}}_{j},$$

where

2 $$\begin{aligned} {\textbf {H}}_{i j}= {\left\{ \begin{array}{ll}{} {\textbf {I}} / 6 \pi \mu R &{} i=j \\ \left( 1 / 8 \pi \mu |{{\textbf {r}}}_{i j} |\right) \left( {\textbf {I}}+{\textbf {r}}_{i j} {\textbf {r}}_{i j} /|{\textbf {r}}_{i j}|^2\right) &{} i \ne j\end{array}\right. }. \end{aligned}$$

Here ${\textbf {I}}$ is an identity matrix, ${\textbf {r}}_{i j}={\textbf {r}}_i-{\textbf {r}}_j$ is the vector from sphere *i* to sphere *j*. At low Re, each swimmer is subject to the force-free condition individually,

3 $$\begin{aligned} \sum _{i=1}^3 {\textbf {F}}_i=\textbf{0}; \;\; \sum _{i=4}^6 {\textbf {F}}_i=\textbf{0}. \end{aligned}$$

The colinear system considered here is also torque-free by axisymmetry. Equations (1)–(3) form a closed system of equations that describes the interaction dynamics of our model microswimmers. In this problem, we choose the radius of the sphere *R* as the characteristic length and use it to scale all lengths in the system. Hereafter we present results only with scaled lengths.

### Reinforcement learning

We employ a deep neural newtork based on an Actor-Critic structure to examine the cooperative behavior of our model microswimmers (Fig. 1b)^41,42^. Both Actor and Critic networks consist of three hidden layers with sizes of 128, 128, and 64 respectively. We implement separated layers between Actor and Critic networks to avoid interference between the two networks. In the current problem, the configurations of the swimmers are discrete and the relative distance between the swimmers is continuous (Fig. 1a,c). This is in contrast with previous studies of locomotion of a single reconfigurable microswimmer that account for either fully discrete or continuous state and action spaces^15,19,20^. Here we extend the deep RL framework in previous studies to tackle state and action spaces with mixed discrete and continuous parameters. We implement a clipped version of the Proximal Policy Optimization (PPO) algorithm^41–43^. See Algorithm 1 in the “Methods” section for the pseudo-code of the PPO algorithm.

At a learning step *n*, the microswimmer pair (learning agent) with observed states $\textbf{S}_n$ performs an action $\textbf{a}_n$ to reach a new state $\textbf{S}_{n+1}$ and obtains a corresponding reward $\textbf{R}_n$ from the environment. In this work, we define our **State, Action, Reward** as follow:

1. **State** $\textbf{S}_n$. The state consists of two parts: The first part corresponds to the discrete configurations of the swimmers’ arm length. Each arm has two possible configurations, either being extended or contracted (Fig. 1c). For two swimmers with a total of 4 arms, the discrete configuration space has a size of $2^4=16$. The second part corresponds to the closest distance *d* between the two swimmers, which is defined as the distance between the closest spheres of the two swimmers (i.e., spheres 3 and 4), and $d =|{\textbf{r}}_4 - \textbf{r}_3 |$ (Fig. 1c). The value of *d* changes continuously during the propulsion of the swimmers, which provides information for the swimmers to adjust their relative position.
2. **Action** $\textbf{a}_n$. The swimmers can perform an action $\textbf{a}_{ij}$ to transition from configuration *i* to configuration *j* (Fig. 1c). Here, we allow both swimmers to actuate at the same time. Namely, each swimmer can choose to actuate one of its arms or to not actuate at each step. We exclude the action where two swimmers both stop at a step. Thus, the action space is discrete with a size of $3^2-1=8$.
3. **Reward** $\textbf{R}_n$. Our learning goal is to maximize the overall net displacement of the two swimmers. To this end, we define the reward $r_n$ at each learning step as the sum of centroid displacement of the swimmer at the back ($D_{B}=\sum _{i=1}^3 D_i/3$) and the centroid displacement of swimmer at the front ($D_{F}=\sum _{i=4}^6 D_i/3$), where $D_i$ denotes the displacement of the sphere $\textbf{r}_i$. We note that the adjustment of *d* is a key component for effective cooperative locomotion, where the swimmers have to maximize the hydrodynamic interactions between each other and avoid collision at the same time. To avoid the collision of the swimmers and to maintain the validity of the Oseen tensor approximation, we introduce a lower bound $d_{lower}$ for *d* between the swimmers. The training episode will terminate when $d \le d_{lower}$. Similarly, we introduce an upper bound $d_{upper}$ for *d* to avoid the swimmer getting too far away, which corresponds to ineffective cooperative locomotion. To ensure a full exploration of the relative distance between the swimmers, we introduce an additional soft bound $d_{soft}$ that is slightly larger than $d_{lower}$. A soft penalization $r_{soft}$ is applied when $d<d_{soft}$ before a sharp termination of learning episode and a hard penalization $r_{terminate}$ are applied at $d \le d_{lower}$. This transition from soft penalization to hard penalization results in a more continuous reward over the learning process, which helps for searching the optimal relative position between the swimmers for effective cooperative locomotion. The same hard penalization $r_{terminate}$ is applied when $d \ge d_{upper}$. As a result, the reward $R_n$ can be expressed as
  4 $$\begin{aligned} \textbf{R}_{n}= {\left\{ \begin{array}{ll} D_{F}+D_{B}, &{} d_{upper}> {d} \ge d_{soft} \\ \ r_{soft}, &{} d_{soft}> {d} > d_{lower}\\ \ r_{terminate}, &{} {d} \le d_{lower}, {d} \ge d_{upper} \end{array}\right. } \end{aligned}$$

Here, $d_{upper}$, $d_{lower}$ and $d_{soft}$ are set as 70, 5 and 6, respectively. $d_{lower}$ and $d_{soft}$ are selected such that the spheres are sufficiently far from each other and the Oseen tensor approximation remains valid (i.e., $d_{soft},d_{lower} \ll 1$). The reward for the buffer region and the termination region are set as $r_{soft}=-0.3$ and $r_{terminate}=-1$. These penalties are set relative to $D_{F}+D_{B}$ which has a typical order of $10^{-1}$.

We limit the length of each training episode to $16 \times 1024 = 16,384$ learning steps, which corresponds to 1024 times of the size of the discrete configuration spaces. This ensures a sufficient number of learning steps for the swimmers to explore the effects of hydrodynamic interactions at different configurations. A discount factor $0 \le \gamma <1$ was introduced to assign a weight to immediate reward over the future reward. We set $\gamma =0.9997$ to ensure farsightedness of the agent. We randomize the initial configurations of the swimmers and the initial closest distance $d_{initial}$ between two microswimmers in each episode to ensure a full exploration of all possible state spaces over training episodes.

We collect all the training information and extract the policy obtained from the training process periodically with a frequency of $2 \times 10^5$ learning steps. The extracted policy is then evaluated in an isolated evaluation environment. Note that the evaluation environment is the same as the training environment. However, there is no additional training performed during the evaluation process. The policy obtained from the training process typically has a probability distribution of various possible actions at a given state of the agent. There are two ways to evaluate the training results, namely stochastic evaluation and deterministic evaluation (see Supplementary Materials for more details). A stochastic policy follows the probability distribution to select the action at each step, whereas a deterministic policy always follows the action with the highest probability. In order to avoid being trapped in undesirable solutions, here we evaluate the extracted policy in a stochastic manner. After sufficient training is performed (i.e., $N_t>10^7$, where $N_t$ is the total number of training step), we observe that continuous training may result in a drop in the performance of the resulting policy. Possible reasons for such a drop in performance are catastrophic forgetting or overfitting^44,45^. In order to select the best policy in the training process, we perform an early stop on training before the performance drops. Such an early stopping has been demonstrated as an efficient way to prevent aggravating policy performance through long-time training in other studies^21,45^.

## Result and discussion

### Cooperative locomotion and gait coordination

We systematically investigate how deep RL achieves an effective strategy for cooperative locomotion. All hyperparameters corresponding to the deep RL algorithm are summarized in the “Methods” section. We train the agent with a control policy $\pi _{\theta }$ to maximize the total displacement of the swimmer pairs. We monitor the training process by considering the moving average of the episodic reward over the last 100 episodes with respect to the total training steps $N_t$ (see Supplementary Fig. S1). We perform an early stop on the training process and select the best model at $N_t=5.4 \times 10^{6}$ steps according to the evaluation result (see Supplementary Fig. S2).

We visualize the selected best policy of cooperative locomotion in an isolated evaluation environment (Fig. 2, Supplementary Movie S1). We place two microswimmers in their fully extended configurations with an initial $d=20$. We note that the policy obtained by RL is insensitive to the choice of initial condition as we have trained the swimmers with different initial conditions during the training process. The policy of cooperative locomotion obtained by the RL comprises two distinct stages, which we refer to as the approach stage (yellow region in Fig. 2a) and the synchronization stage (pink region in Fig. 2b). The closest distance *d* first gradually decreases in the approach stage until it reaches a minimum value close to $d_{soft}$, after which *d* oscillates periodically in the synchronization stage (Fig. 2a). We note that *d* will still be adjusted slightly during the synchronization stage. After a prolonged simulation, *d* will eventually get almost equal to $d_{soft}$ (see Supplementary Fig. S3). The transition from the approach stage to the synchronization stage is classified by a switch in the pattern of *d* and hence a switch in the locomotory gaits of the swimmers. We consider it to be a complete transition when a new pattern of *d* repeats for 5 gait cycles. The occasional disturbances in *d* in the two stages are effects due to stochastic evaluation. In the approach stage, the swimmer at the back has substantially larger net displacement ($D_B$, red line in yellow region of Fig. 2b) than the swimmer at the front ($D_F$, blue line in yellow region of Fig. 2b), therefore the swimmer at the back will “approach” the swimmer at the front. In the synchronization stage, the two swimmers have essentially the same net displacement, as indicated by the same slope and the same periodic pattern of $D_B$ and $D_F$ in the pink region of Fig. 2b.

Now we elaborate how the AI-advised policy achieves effective cooperative locomotion by analyzing the details of the locomotory gaits of the swimmers at the two stages (Fig. 2c,d). An effective cooperative locomotion is subject to two major challenges: first, the swimmers have to adopt a strategy to approach each other and swim together, while not getting too close to collide with each other; second, the swimmers have to finely coordinate their locomotory gaits to exploit hydrodynamic interactions to maximize their overall propulsion. Here we show that these two challenges are indeed tackled properly by the AI-advised locomotory gaits of the swimmers at the two stages correspondingly. During the approach stage (yellow region in Fig. 2a,b, Supplementary Movie S2), the swimmer at the back swims in N-G strokes (Fig. 2c, red spheres, and approaches the swimmer at the front, whereas the swimmer at the front only exhibits forward propulsion occasionally, as can be seen from the relatively flat slope of $D_F$ in Fig. 2b. Most of the time the swimmer at the front does not perform any motion or performs reciprocal motions that lead to zero net self-propulsion at low Re (Fig. 2c, blue spheres)^29^. As a result, the swimmer at the front “waits” for the swimmer at the back to catch up. After the swimmers are in sufficiently close proximity (i.e., minimum $d \approx d_{lower}$), the swimmers start to synchronize their locomotory gaits. In the synchronization stage (Supplementary Movie S3), the swimmers propel forward with N-G strokes, where there is a constant phase difference in the N-G strokes adopted by the two swimmers. Namely, the swimmer at the front has a delay of 1 actuation step in the N-G strokes compared to the swimmer at the back (Fig. 2d). The synchronized swimmers maintain a fixed range of *d* and propel with the same overall displacement (pink region in Fig. 2a,b).

**Figure 3:**
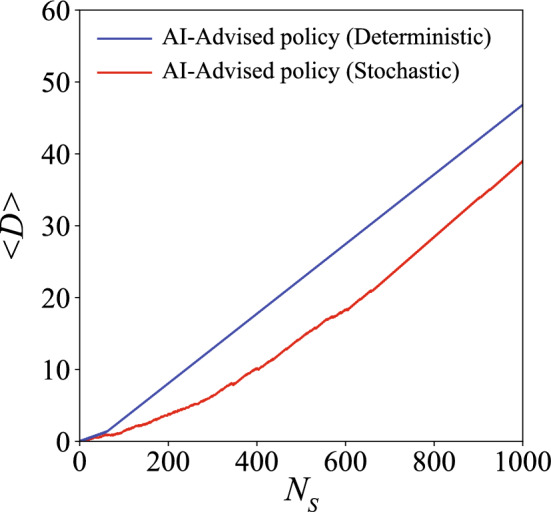
Comparison of average displacement $\langle D \rangle$ of the swimmer pair between the deterministic policy and the stochastic policy. The blue line and the red line denote the results for the deterministic policy and the stochastic policy, respectively.

**Figure 4:**
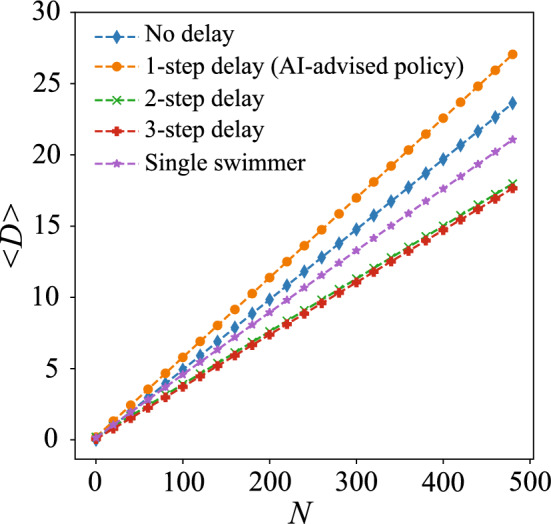
Comparison of average displacement $\langle D \rangle$ of the swimmer pair with prescribed N-G strokes and different phase mismatches. The colored lines denote the cases with different delays in N-G strokes of the swimmer at the front, including the cases of no delay (blue line), 1-step delay (orange line, AI-advised policy), 2-step delay (green), 3-step delay (red). Displacement of a single swimmer following N-G strokes (purple line) is added to benchmark the performance of cooperative locomotion of different prescribed locomotory gaits.

We remark that in addition to the best model, the RL algorithm also acquires several suboptimal models throughout the training process, as can be seen from the local peaks in the average episodic rewards (Supplementary Fig. S1). These suboptimal models also exhibit locomotory gaits with approach and synchronization stages similar to the best model. However, these suboptimal models fails to adjust *d* properly in the approach stage and results in minimum *d* being larger than $d_{soft}$ in the synchronization stage. Thus the suboptimal models fail to fully exploit hydrodynamic interactions between the swimmers for effective cooperative locomotion, leading to smaller episodic rewards. We note that the implementation of the soft bound $d_{soft}$ in the reward in Eq. (4) is a key to obtain the best model with the minimum value of *d* being close to the lower bound $d_{lower}$ in the synchronization stage. If the soft bound $d_{soft}$ is not implemented, the policies obtained by RL will end up with a much larger *d* in the synchronization stage and the swimmers will fail to fully exploit the hydrodynamic advantage from their interactions.

### Comparison between deterministic and stochastic policies

The AI-adivsed policy has successfully demonstrated how a swimmer pair achieves cooperative locomotion. Albeit being more robust in avoiding undesirable solutions, the policy achieved by stochastic evaluation has introduced random noises in the locomotory gaits. Here we investigate how such a stochasticity influences the overall locomotory performance by comparing the stochastic policy and the deterministic policy. In the deterministic policy, the swimmer at the front does not generate any net propulsion and waits for the swimmer at the back to get close in the approaching stage, instead of exhibiting random motions with a small overall displacement. After the swimmers get sufficiently close (i.e., $d \sim d_{soft}$), the swimmers enter the synchronization stage and propel with N-G strokes, where the strokes of the two swimmers have the same constant phase shift as the stochastic policy. We compare the average displacement of the two swimmers, i.e., $\langle D \rangle =(D_B+D_F)/2$ for the deterministic policy and the stochastic policy (Fig. 3, Supplementary Movie S4). The deterministic policy outperforms the stochastic policy at a short time scale ($N_s < 500$) as the swimmers have no random actions and enter the synchronization stage earlier. However, both policies perform approximately equal at a long time scale where they all enter the synchronization stage ($N_s > 500$). Nevertheless, the stochastic policy has a more stable performance during training, while the determinsitic policy can possibly be trapped in undesirable solutions with unexpectedly small net displacement during the training process.

### Comparison with other synchronized locomotory gaits

Our AI-advised policy suggests there exists a phase difference between the synchronized N-G strokes of the swimmer pair that maximizes the displacement. To further investigate how the phase difference influences the cooperative locomotion, we prescribe the locomotory gaits of the swimmer pair with N-G strokes with different mismatches in phase (i.e., different delays in actuation step for the swimmer at the front) and compare their locomotory performance. For a fair comparison, all prescribed gaits have the same minimum closest distance $d=6$. We measure the locomotory performance of different prescribed gaits by the average displacements of the two swimmers, i.e., $\langle D \rangle$. The evolution of $\langle D \rangle$ for N-G strokes at different phase mismatches are displayed in Fig. 4 and Supplementary Movie S5. Our results demonstrate that the AI-advised locomotory gait (Fig. 4, orange dashed line) has indeed learnt the most effective phase mismatch of N-G strokes for cooperative locomotion among all mismatches considered. We also benchmark the locomotory performance of the prescribed locomotory gaits with a single swimmer with N-G strokes (Fig. 4, purple dashed line). We note that not all the synchronized N-G strokes outperforms the locomotory performance of a single swimmer. For the cases where the swimmer at the front has a delay of 2 or 3 steps in its N-G strokes (Fig. 4, green and red dashed lines), the synchronization of N-G strokes of the swimmer pair can be counterproductive, leading to a locomotory performance worse than that of a single swimmer alone. In contrast, the synchronized N-G strokes obtained by RL improves the propulsion speed by ~ 25% compared to the N-G strokes of a single swimmer. Here we demonstrate how RL successfully searches for effective locomotory gaits to achieve cooperative locomotion.

## Conclusion

In this work, we present the first use of deep RL to empower a pair of microswimmers to cooperate for enhanced locomotion at low Re. The AI-advised policy of cooperative locomotion can be distinguished in two stages: an approach stage where the swimmers adjust their relative distance to get in close proximity for increasing their hydrodynamic interactions, followed by a gait coordination that optimizes the hydrodynamic interaction between the swimmers in a synchronization stage. The self-learning of this AI-advised policy involves the consideration of state and action spaces that include both discrete and continuous components. While fully continuous state and action spaces can be considered as in Refs.^15,18–20^, this may significantly increase the number of learning steps to search for the optimal solution. We consider an axisymmetric, colinear configuration in this work as arguably the simplest model problem to explore the cooperative behavior of smart artificial microswimmers, while the deep RL framework here also applies to more general configurations. Subsequent works will build on the framework to investigate the cooperative behaviors of increased numbers of microswimmers with more complex spatial arrangements, probing the probable emergence of pattern formation^23,46^ among smart artificial microswimmers, analogous to collective behaviors of fish, bird, and microorganisms observed in nature^47–49^. The consideration of non-colinear configurations may require more complex swimmer models that allow combined translational and rotational motion^20^ to exhibit effective cooperative behaviors. Larger state and action spaces will have to be set up for the learning agent to incorporate these additional complex maneuvers. We envision that the RL approach here would be particularly relevant for coordinating a group of artificial microswimmers to perform collective microrobotic tasks that require fine adjustments of spatial locations within the group^50,51^. Here we reward the microswimmers for maximizing their overall net displacements; future works will explore the design of other reward functions to empower microswimmers to cooperate for different tasks such as chemotaxis^19,52^. Lastly, we also remark on the potential use of the centralized training and decentralized execution approach in multi-agent RL, which capitalizes on access to the full state and information during the training phase while addresses the challenge of individual agents not having access to the full state during the executive phase. Such an approach has been shown effective in different real-world applications^53,54^ and may be considered as an alternative approach in future works.

To conclude, we have presented the first use of RL to achieve effective cooperative locomotion of artificial microswimmers endowed with AI. This proof-of-principle demonstration opens up new opportunities towards intelligent autonomous manipulation of multiple microrobots, laying the groundwork for their future biomedical and environmental applications^50,55,56^.

## Methods

### PPO algorithm

We utilize a PPO clipped version^41^, and calculate the advantage through Generalized Advantage Estimation^43^. The pseudo-code for the PPO clipped version is shown in Algorithm. 1. See Supplementary Materials for more details about the deep RL algorithm.
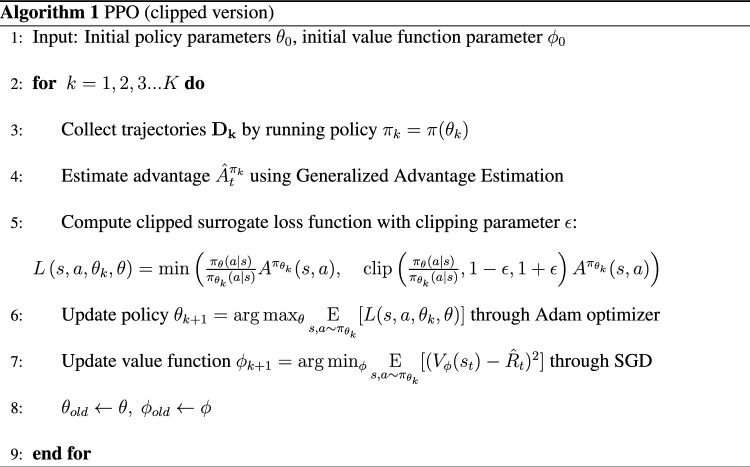


### Hyperparameters

Here we elaborate on the hyperparameter settings we have used for training the cooperative locomotion of the microswimmer pair. The hyperparameters are tuned based on the training performance of different learning trails. It is conceivable that a better model can be learnt through systematic parameter tuning. We do not perform hyperparameter optimization due to the cost of computational expense. The hyperparameters are shown in Table 1.

**Table 1:** Hyperparameters for deep reinforcement learning.

Name of hyperparameter	Value	Description
Learning rate	0.0001	The learning rate used by Gradient descent
Neural network update frequency	16,384	Number of environment steps to run for each neural network update
Neural network architecture	Actor:128,128,64 Critic:128,128,64	Size of three hidden layers for the Actor-Critic network
Batch size	256	Number of training cases to be computed at each stochastic gradient descent (SGD)
Epoch	10	Number of time for the whole training cases computed at SGD
Discount factor	0.9997	Factor for computing discounted future reward
Clip range	0.2	Clipping parameter for PPO algorithm
Target KL divergence	N/A	Limit the difference between current policy and new policy

## Supplementary Information

Supplementary Movie S1.

Supplementary Movie S2.

Supplementary Movie S3.

Supplementary Movie S4.

Supplementary Movie S5.

Supplementary Information.

## Data Availability

The data that support the findings of this study are available from the corresponding author upon reasonable request.

## Code availability

The codes that support the findings of this study are available from the corresponding author upon reasonable request.
