# Supplementary material for: Learning to cooperate for low-Reynolds-number swimming: a model problem for gait coordination
Source: Sci Rep. 2023 Jun 9;13:9397. doi: 10.1038/s41598-023-36305-y (PMC10256736; doi:10.1038/s41598-023-36305-y)
Supplement: Supplementary file 6 — Supplementary Information. [file 41598_2023_36305_MOESM6_ESM.pdf]

Supplemental Materials for "Learning to cooperate for low-Reynolds-number swimming: A model problem for gait coordination"

Yangzhe Liu[1], Zonghao Zou[2], On Shun Pak[3] and Alan C. H. Tsang[1*]

I. DESCRIPTION OF SUPPLEMENTAL MOVIES

Movie S1: Two 3-sphere microswimmers learn to swim cooperatively via deep reinforcement learning. Microswimmers switch from the approach gait to the synchronization gait after 500 steps. The red dashed line denotes the midpoint between the closest spheres of the two swimmers.

Movie S2: A slow motion video of the approach gait.

Movie S3: A slow motion video of the synchronization gait.

Movie S4: Comparison of the deterministic policy and the stochastic policy. The red dashed lines for the swimmer pairs denote the mid-points of their closest spheres.

Movie S5: Comparison of cooperative locomotion of the swimmer pair with prescribed N-G strokes and different phase mismatches. The red dashed lines for the swimmer pairs denote the mid-points of their closest spheres. The red dashed line for the single swimmer is placed at a location of 3 dimensionless units from the front sphere, which corresponds to half of the minimum closest distance between a swimmer pair.

II. TRAINING AND EVALUATION

In this section, we present the progressive improvement of training results during reinforcement learning and discuss how we select the best model. We first calculate the moving average of the reward over the last 100 training episodes, which we define as $\overline{r}_E$. We then monitor how $\overline{r}_E$ varies during the training process (Fig. S1). It can be seen that $\overline{r}_E$ first increases and then fluctuate with $\overline{r}_E > 1000$. We note that this fluctuation is due to the fact that the search for optimal relative distance d between the swimmers for the synchronization stage is a complicated task for the learning process. Finding a better d close to the lower bound d_{lower} defined in main text Eq. (4) will usually result in a slight improvement in the locomotory performance. However, the search of better d would require exploration of state spaces with a much larger perturbation in the reward of a given episode, leading to fluctuation in $\overline{r}_E$.

We extract the model periodically and evaluate it in a separate environment to directly examine the training progress of the RL model. We extract the model periodically with a frequency of 2×10^5 training steps. We evaluate the extracted models by simulating them stochastically with a fixed number of steps (16×1024 steps). In our model evaluation, we always evaluate the model stochastically to obtain the episodic reward of the model. By stochastic evaluation, here we mean that the policy trained from PPO is stochastic $\pi_\theta(a \mid S) = \mathbb{P}[a \mid S; \theta]$. For a given stochastic policy, there is a probability distribution for selecting an action a at a given state S. This is in contrast to the deterministic evaluation, where the policy always follow an action a with the largest probability for a given state S, instead of following the probability distribution for selecting an action. Thus, stochastic evaluation always has a slightly different episodic reward, and deterministic evaluation always has the same episodic reward. Here we average the episodic reward of 3 stochastic evaluations for each model and summarize the results in Fig. S2. We collect the average episodic rewards and select the model with the best result, which we highlighted with the red circle in Fig. S2.

III. PROLONGED SIMULATION OF THE AI-ADVISED POLICY

For a prolonged simulation of the AI-advised policy, the closest distance d in the synchronization stage will slightly decrease over increased N_s, but at a much slower rate compared to the approaching stage. The minimum d will eventually get very close to d_{soft}. The change in d for the prolonged simulation is presented in Fig. S3.

---

* [1] Dept. of Mechanical Engineering, The University of Hong Kong, Hong Kong, alancht@hku.hk; [2] Sibley School of Mechanical and Aerospace Engineering, Cornell University, Ithaca, NY 14850, USA; [3] Dept. of Mechanical Engineering, Santa Clara University, Santa Clara, USA

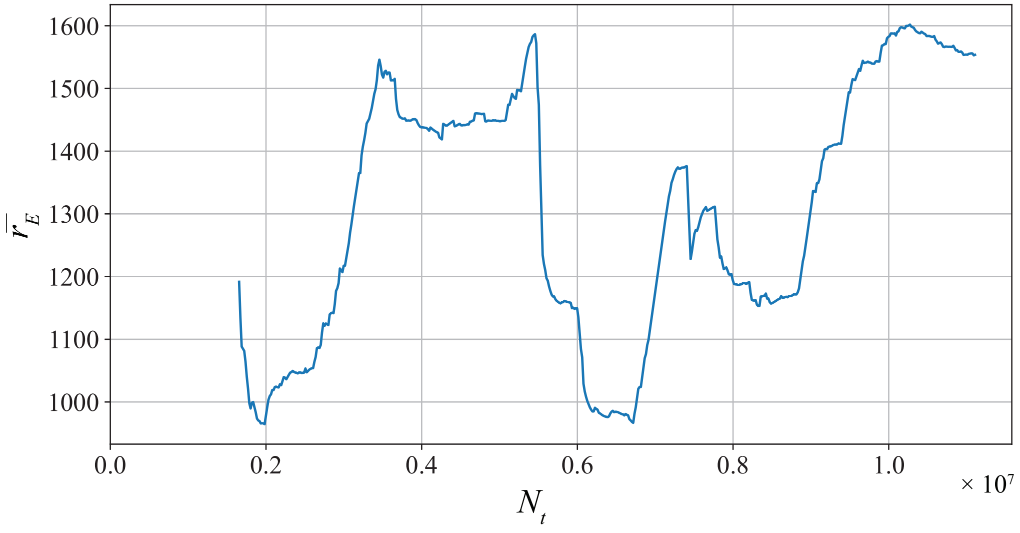

FIG. S1. The average episodic rewards are collected by monitoring the moving average of rewards over the last 100 episodes.

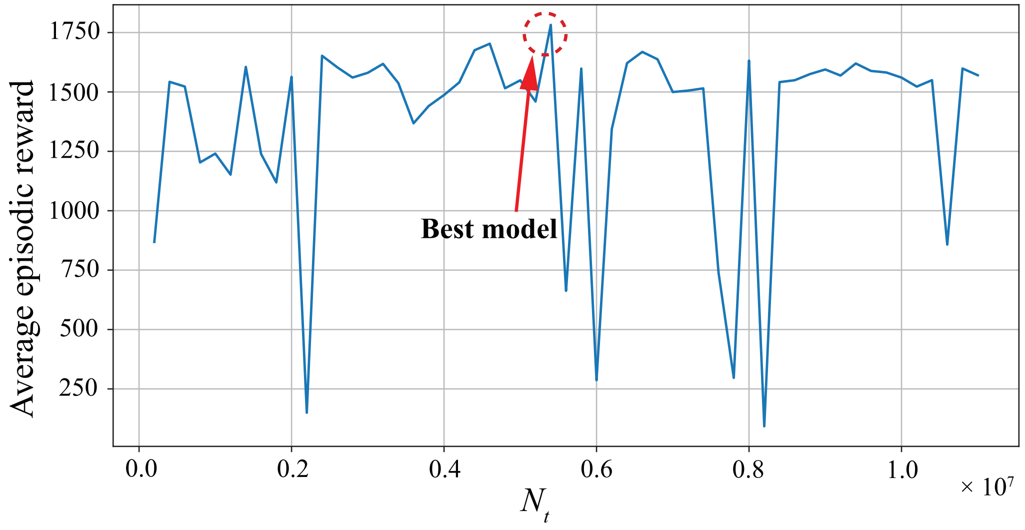

FIG. S2. Evaluation result of the training process by averaging episodic reward of 3 stochastic evaluations.

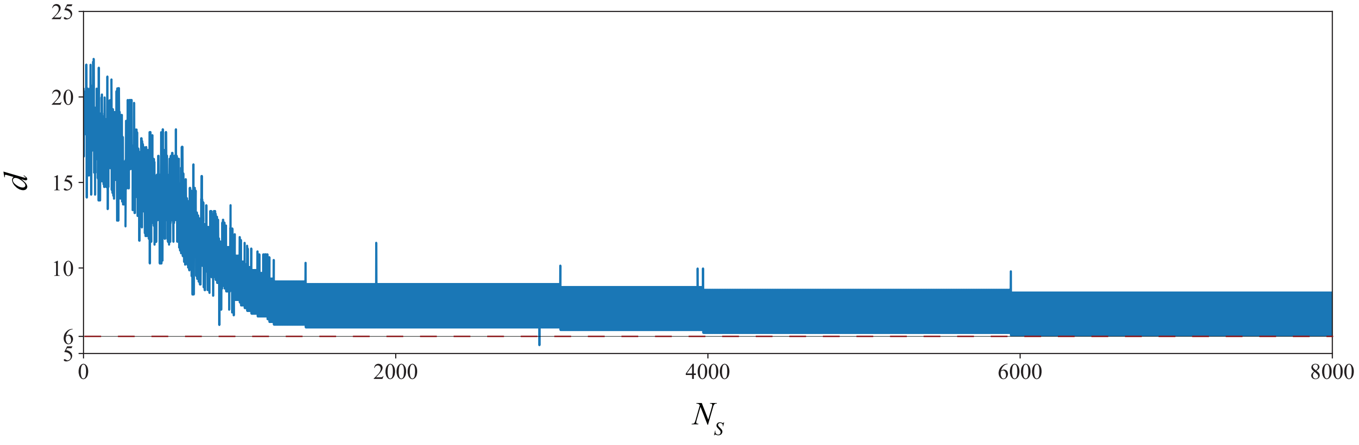

FIG. S3. Prolonged simulation of the AI-advised policy. The red dash line denotes the the value of $d_{soft} = 6$.
